# A qualitative evaluation of scalpel skill teaching of podiatry students

**DOI:** 10.1186/s13047-017-0202-9

**Published:** 2017-05-02

**Authors:** Ryan S Causby, Michelle N McDonnell, Lloyd Reed, Caroline E Fryer, Susan L Hillier

**Affiliations:** 1 Sansom Institute for Health Research, University of South Australia, GPO Box 2471, Adelaide, SA 5001 Australia; 2 Alliance for Research in Exercise, Nutrition and Activity, University of South Australia, Adelaide, SA 5000 Australia; 3 School of Clinical Sciences, Queensland University of Technology, Kelvin Grove, QLD 4059 Australia

**Keywords:** Clinical education, Manual clinical skills, Skills acquisition, Low-fidelity simulation, Qualitative research, Scalpel skill teaching

## Abstract

**Background:**

Degrees in health disciplines need a balance of theoretical knowledge and sufficient clinical practice to meet registration requirements, in particular those requiring specialist skills such as the use of scalpels and other small instruments, such as podiatry. However, despite this requirement there is a scarcity of literature and research to inform teaching of these particular manual clinical skills. Therefore, the aims of this study were to determine the current approaches being used to teach manual skills, in particular scalpel skills, in university podiatry programs in Australia and New Zealand, and to explore what issues, challenges and innovations exist.

**Methods:**

A qualitative study, consisting of semi-structured interviews with staff at eight university podiatry programs in Australia and New Zealand was undertaken to determine how these skills are taught and evaluated, and how poor performers are managed. A conventional content analysis technique was used to analyse and code interview data, with the resultant categories reported.

**Results:**

Approaches to teaching manual clinical skills, in particular scalpel skills, appear to be consistent between university programs in Australia and New Zealand in utilising didactic-style content, demonstration, physical practice on inanimate objects and real skin, and often the use of supplementary audio-visual material. The main reported differences between programs were in methods and processes of practice, with controversy regarding the use of inanimate objects versus real skin for practice.

**Conclusions:**

Despite a lack of research and literature surrounding this topic, the approach to teaching is relatively consistent between programs with greatest disparity being the structure and duration of practice. Key issues for teaching staff in teaching manual skills were students’ clinical exposure, motivation, levels of anxiety and dexterity.

## Background

Health disciplines are relatively unique in their needs with respect to tertiary training. They need to provide a sound theoretical knowledge base from which to base their clinical decisions, whilst at the same time having sufficient motor skills in order to provide some of the required management strategies. This includes manipulation of many small instruments. The consequence of insufficient skill can be of great detriment. An example of this is the use of scalpels in podiatry. Podiatrists regularly use scalpels to debride plantar callus (built up from pressure), remove non-viable tissue from wounds, enucleate corns and manage problematic nails. However, there are a number of challenges with teaching such skills. For example, these skills need to be taught in a relatively safe, and where possible, controlled environment. Teaching is often much more time and labour-intensive. Furthermore, in many countries there are increasing student numbers and subsequent pressure on clinical resources, including limited clinical placement options [1, 2]. This has prompted research and development into many alternatives, such as virtual-reality trainers, which often come at a rather substantial cost. However, despite this, there is very little robust documented evidence regarding how such skills are currently taught, and any associated pros and cons with particular methods. Naturally, we expect that this could differ substantially between countries and disciplines. Therefore, we see a benefit in investigating how such skills are taught relative to these in order for a cross-pollination of ideas. In this instance, the teaching of scalpel skills within the podiatry programs from Australia and New Zealand were investigated.

Therefore, the aims of this study were:

1. To determine the current approaches being used to teach manual skills, in particular scalpel skills, in university podiatry programs in Australia and New Zealand,
2. To explore what issues, challenges and innovations exist with the teaching of scalpel skills.

## Methods

Ethical approval was obtained from the Human Research Ethics Committee at the University of South Australia. A series of semi-structured interviews was chosen as the most appropriate method in order to allow greater exploration of the issues surrounding a topic with a paucity of evidence, whilst providing a structure with which to undertake an appropriate qualitative analysis. Semi-structured interviews allow interviewers to prepare predominantly open-ended questions ahead of time, to be delivered in a consistent order, but allows the flexibility to follow topical trajectories in the conversation which may stray from the guide when appropriate [3, 4].

The interview guide was developed in conjunction with the research team (two podiatry academics and two non-podiatry health researcher academics) and piloted prior to recruitment, on two University of South Australia Podiatry teaching staff not involved in its development for content validity, and to evaluate feasibility and utility. No changes were made as a result of piloting.

### Recruitment

A purposeful maximum variation sampling strategy was employed [5]. We targeted Podiatry program staff best positioned to provide a global program overview and knowledge of curricula development, and another nominated staff member directly involved in skill teaching to provide a rich diversity of opinion and strengthen the credibility of the data [6]. An e-mail was sent to the relevant staff at the eight universities in Australia and New Zealand offering podiatry programs at the time of the study (2011/2012), inviting them to participate in a semi-structured interview.

### Data collection

**Fig. 1:**
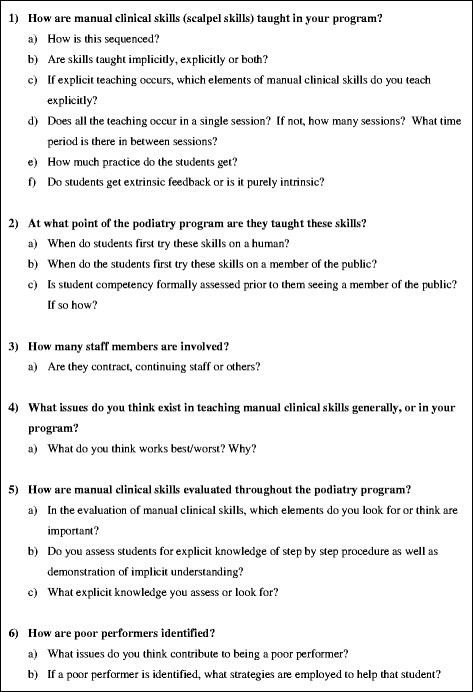
Semi-structured interview questions

Once a completed consent form was returned, an interview time was negotiated to occur face-to-face, or via telephone for participants located interstate to the researcher. The primary researcher (RC) conducted all interviews using a semi-structured interview guide (Fig. 1) to gain data relevant to the research aims. Questions were arranged under the following topic areas: how skills are taught; evaluation of skills; and, management of poor performers. Information regarding the participants’ experience teaching outside of Australia or New Zealand was collected, and reported where relevant, to provide further insight to possible responses but not disclose their identities. For example, participants who have taught or worked outside of Australia or New Zealand may provide insight beyond the local context.

### Data analysis

A conventional content analysis was used [7] as a systematic and objective means of describing and quantifying the studied phenomena [8–10] whilst maintaining a high level of flexibility with respect to research design [10, 11]. A quantitative content analysis was used to determine program structure and teaching methods to address research aim one. Detail regarding course structure and participant reporting information was also elicited from the relevant university webpages and via the Australian and New Zealand Podiatry Accreditation Council (ANZPAC) webpage. An inductive qualitative content analysis was used to explore the data in-depth regarding teaching issues, successes and strategies to address research aim two [10]. Care was taken to ensure meaning, intention and context were elicited [9]. An analysis of manifest content, and not latent content (such as non-verbal communication), was employed [9].

Each transcribed manuscript was read independently by the primary researcher (RC) and another researcher (MM) who was not involved in the interview or transcription processes. Open coding of each transcript was undertaken independently by both researchers and transferred to a spreadsheet [7]. Meaning units were determined from interview content relating to the same central meaning [6]. Each meaning unit was then labelled with a code by the researcher that summarised the meaning of the data. Examples of this can be found in Table 1. After initial coding, the two researchers independently categorised the coded data and then met to discuss the coding and categorisation and ensure consensus. Coding and categorisation continued until all meaning units were incorporated into categories [6].

**Table 1:** Examples of codes, sub-categories, categories and theme as part of content analysis

Meaning Unit	Code	Sub-category	Category	Theme
*“If too many educators then (students) find that too confusing to go from educator to educator”*	Student confusion in teaching	Consistency in teaching	Teaching related issue	Issues
*“Practising on inanimate objects works well”*	Successful use of inanimate object	Inanimate objects	Teaching related success	Successes

Trustworthiness of the data collection and analysis process, incorporating credibility, dependability and transferability, was ensured in several ways. To ensure credibility two participants were interviewed from each site. To ensure dependability, interviews were recorded and data analysis was undertaken independently by two researchers with diverse clinical backgrounds. Details about the primary researcher’s qualifications, experience and perspective are reported in addition to participant characteristics to allow scrutiny of their potential biases and influence on data collection and interpretation [5]. Transferability of the study was supported by detailed reporting of the context, selection and characteristics of participants and the data collection process [6].

Participant and university ‘codes’ were randomly allocated to present the data whilst allowing participant quote to remain anonymous.

## Results

Interviews were conducted between November 2011 and January 2012 with 16 experienced educators; two from each of the eight university podiatry programs in Australia and New Zealand. The nominated staff members participating in the interviews were full-time salaried or sessional staff experienced in teaching manual clinical skills including scalpels skills. Participants had an average (SD) of 20.5 (±8.9) years of experience. Three educators who participated in the study had also taught in the United Kingdom. The primary researcher who conducted the interviews and reported the study was an educator with 10 years’ experience in teaching scalpel skills (RC). The second researcher was a physiotherapist and neuroscience researcher with experience both clinically and as an educator who could challenge discipline-specific assumptions in the analysis (MM).

**Fig. 2:**
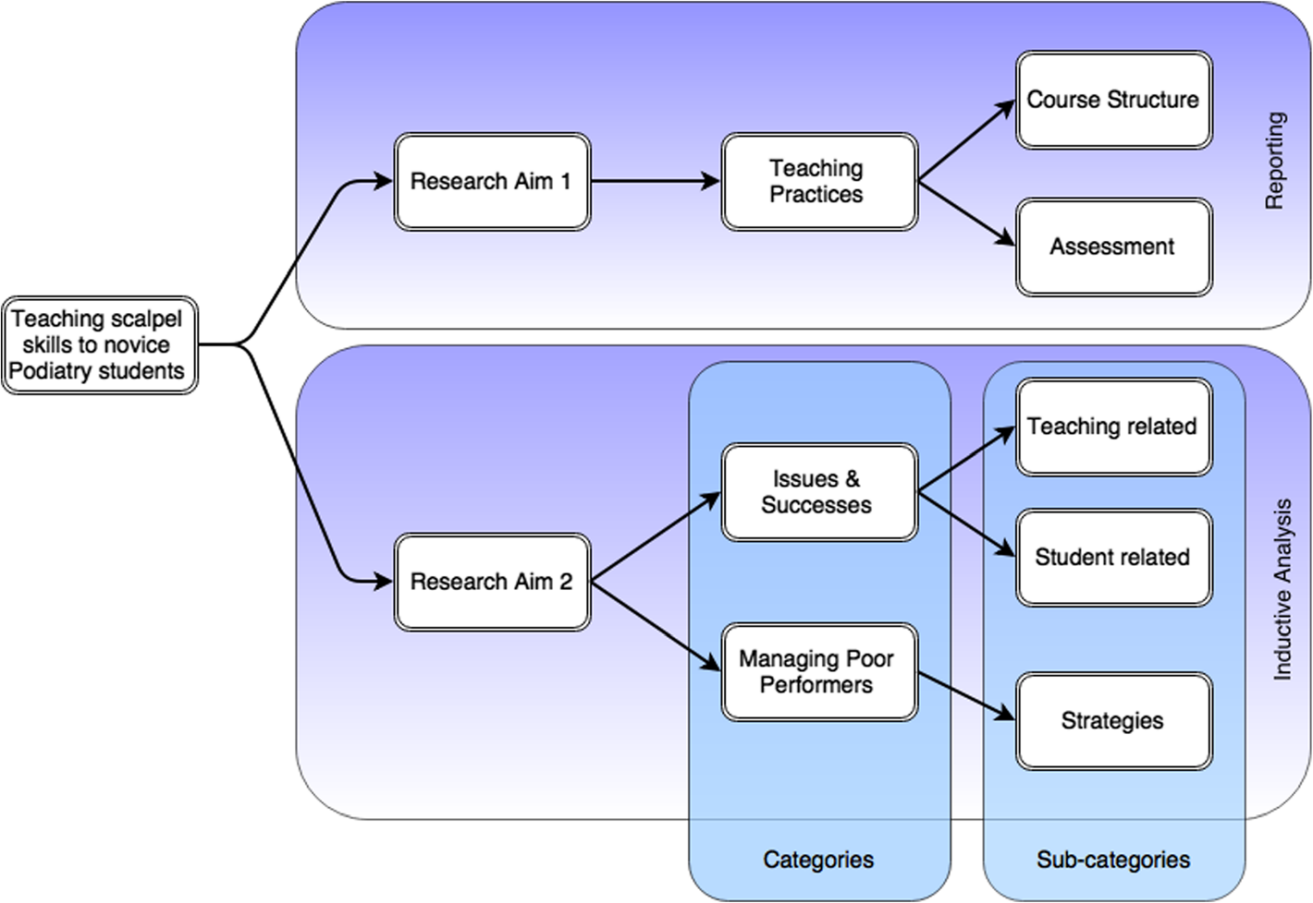
Resulting structure of content analysis

Two categories and three subcategories were determined from the overall analysis as shown in Fig. 2. Study findings have been reported under the relevant category title.

### Teaching practices

Quantitative findings regarding the structure and methods of teaching manual clinical skills to novice podiatry students are reported as two distinct areas: ‘Program structure’, and ‘Assessment’ of manual clinical skills competency.

#### Program structure

There was diversity in the reported content and structure of university programs offered around Australia and in New Zealand (Table 2). Programs ranged from a 3-years Bachelor degree to graduate entry Masters and Doctor of Podiatric Medicine programs.

**Table 2:** Structure of podiatry programs for teaching scalpel skills in Australia and New Zealand

University	Degree	Taught	First Patient Contact	Duration between (Semesters)	Low fidelity simulation methods	Additional Motor practice arrangements^a^
1	3 year Bachelor	1^st^ Year, 2^nd^ Semester	2^nd^ Year, 1^st^ Semester	1	Wax impregnated materials	Take home/Allowed to take home but not encouraged/University clinic – additional allowed
2	3 year Bachelor	2^nd^ year, 1^st^ Semester	2^nd^ year, 1^st^ Semester	0	Wax impregnated materials	Allowed to take home but not encouraged
3	4 year Bachelor	2^nd^ year preclinical	2^nd^ year 1^st^ Semester/2^nd^ Semester	0–1	Wax impregnated materials and other inanimate objects	Take home/Not allowed to take home
4	4 year Bachelor/3 year GE Doctor of Podiatric Medicine	1^st^ Year, 2^nd^ Semester	2^nd^ Year, 1^st^ Semester	1	Wax impregnated materials	Not allowed to take home/Additional university clinic experience
5	4 year Bachelor	2^nd^ Year, 1^st^ Semester	2^nd^ Year, 1^st^ Semester	0	Wax impregnated materials and other inanimate objects	Take home
6	4 year – Bachelor & Masters (double degree)/2 year GE Masters	2^nd^ Year, 1^st^ Semester	3^rd^ Year, 1^st^ Semester	2	Wax impregnated materials and other inanimate objects	Allowed to take home but not encouraged/Not allowed to take home
7	4 year Bachelor/2.5 year GE Masters	2^nd^ Year, 1^st^ Semester	2^nd^ Year, 2^nd^ Semester	1	Fleecy web only	Only university clinic/Additional university clinic experience
8	4 year Bachelor - Masters (double degree)	2^nd^ Year, 2^nd^ Semester	3^rd^ Year, 1^st^ Semester	1	Wax impregnated materials and other inanimate objects	Allowed to take home but not encouraged/Not allowed to take home

^a^ *Note:* discrepancies occurred due to varying responses amongst participants

#### When scalpel skills are taught

The timing of introduction to scalpel skills for students varied from the second semester of first year to the second semester of second year. The timing of when students first used a scalpel on a member of the public also varied, again from second semester of first year, to the first semester of third year. The duration between first learning scalpel skills and first seeing a member of the public was as short as 4 to 5 weeks and as long as almost a year (Table 2).

#### How scalpel skills are taught

All eight programs taught scalpel skills in clinics or skills laboratories with seven of the programs reporting this was preceded with the teaching of theoretical aspects either large lecture format, tutorials or via written or recorded means (such as written manuals or DVDs).

Four of the programs reported using video, DVD or alternate multimedia in addition to live demonstration of the technique. At least two of the programs reported providing a copy, or making the video available, for students to view at their convenience. One program also used video streaming to allow multiple students to watch a live demonstration on screens if required.

One program reported teaching students to use the scalpel handle without a blade attached first, to allow them to develop the technique initially without the risk of damage to themselves. More commonly, a structured approach was used incorporating demonstration followed by part task training, constituting practice on inanimate objects including oranges, avocadoes, soap, cork, alginate, potatoes, fleecy web, wax, candles, chicken wings or even blue tac® in a peg. Subsequent progression of training was often to low fidelity simulation such as to undertake debridement of wax from fleecy web or other backing (wax impregnated materials), attached to a colleague’s foot or in one instance where only the fleecy web was attached to the foot. One program extended this to include embedding small seeds within the wax to replicate corns (Heloma Dura) and another included rubberised feet to increase fidelity during the early stages of practice in a reasonably safe manner. Table 2 includes the approach and objects described for use within each of the university programs.

Participants from five programs reported that students would then advance to debriding actual callus from fellow students. However, it was reported that this was not always possible as many of the students were too young to have developed significant skin pathology.

The final step in training of scalpel skills for the students was to treat members of the public within the University clinic setting (Table 2), using scalpels on a case-by-case basis when required. All but one of the programs managed a university-based clinic as part of their program. The remaining program used a university-staffed clinic based in a public health institution.

The greatest diversity in teaching practice of scalpel skills between programs was the duration and structure of clinical practice arrangements, including scalpel use. Six of the programs reported using a large tutorial block or skills laboratory. This block could be dedicated to scalpel practice once per week and then students moved on to an alternate clinical skill in subsequent weeks (massed practice), compared to another program which reported having short sessions dedicated to scalpel practice prior to patient clinics which were repeated weekly but students were introduced to other clinical skills as the weeks progressed (distributed practice).

Students often undertook further motor practice, additional to core teaching, via a number of methods. This included taking home instruments, and being allowed additional and optional time within the university clinic environment both with and without accessibility to staff. It was noted that in some programs, students were required to purchase their own instruments for use in the clinic, whilst in others, the universities provided instruments. It was unclear on how this impacted on whether or not they were allowed to practice at home. However, there was quite a diversity of opinions from interview participants, even within a single program, about whether students should be allowed to take instruments home to practise on family members or friends. Whilst three of the programs encouraged this, others forbade the practice and the remaining programs fell somewhere in the middle (see Table 2).

Before commencing practice on patients, four programs used a mentoring system with more experienced students (third or fourth year). This allowed the novice student either time during a formal clinic period observing (most common) or occasionally undertaking minor treatment processes with peer support. A majority of the programs also reported having students work in pairs during initial practice and when the students started with real patients in the clinic setting.

#### Assessment

Participants expressed strongly that the capability of students with respect to manual clinical skills needed to be assessed practically, which was difficult to do through only written or verbal means. For four of the programs this was achieved through the use of competency checklists (Table 3). For some programs this was achieved on an ongoing basis, where students were required to get a particular competency signed off by a supervising clinical educator one or more times throughout the specified clinical period. Three programs described once-only competency exams where students demonstrated particular techniques or skills at a set point in time during the subject. For many of these programs the competency checklist or exam was a required milestone which students needed to satisfactorily pass in order to progress to practice on the public. Finally, all programs used an Objective Structured Clinical Exam (OSCE) or patient examination as a component of the assessment (Table 3). In the case of the patient exams this meant that examiners and students were often *“…at the mercy of what turns up at the time”* (Participant 11).

**Table 3:** Reported methods for assessing manual clinical skills (scalpel)

University	Competency checklist	Patient exam	OSCE	Ongoing clinical	Reflective journal/logbook
1			✓	✓	
2	✓	✓	✓		
3	✓		✓		✓
4		✓		✓	✓
5			✓	✓	
6	✓		✓	✓	
7		✓		✓	
8	✓		✓	✓	

*OSCE* Objective Structured Clinical Examination

Assessment methods and ongoing clinical observation were recognised by participants to play an important role in identifying poor performers. An exception to this was one participant who felt that judging the particular point of competency was difficult and therefore not formally evaluated.

*“…because the skill learning (sic) is developed over time it is hard to have a black and white point to say that they are competent and therefore there isn’t a lot of formal assessment going on”* (Participant 2).

Another participant (Participant 12) reported that they did not undertake any formal assessment until the end of semester, relying on ad hoc feedback and admitted that students who picked up a poor technique were occasionally ‘missed’ and not picked up until the examination stage.

In order to understand in more depth the process of scalpel skill learning to address research aim two of the study, participants were asked for their perspective on what worked well or didn’t work well within their program. Two categories resulted from the inductive coding process: the first involved ‘Issues and successes’ with respect to teaching structure, and the second was ‘Managing poor performers’ (Fig. 2).

### Issues and successes

Issues and successes in the teaching of manual clinical skills to podiatry students were sometimes identified in opposing contexts by different participants. For example, one participant identified an issue such as ‘teaching scalpel skills being quite labour-intensive’ (Participant 3), whilst another identified the same issue as a success due to ‘having low student to staff ratios’ (Participant 7). Where possible these themes have been grouped accordingly and discussed in both contexts. In some cases, opposing opinions were expressed. For example, even within programs some participants felt that the use of inanimate objects was a particularly successful strategy, whilst others expressed that inanimate objects were an inadequate replacement for practice on a real foot. There were two main sub-categories identified for ‘Issues and successes’ in teaching manual clinical skills, in particular scalpel skills, to podiatry students: Teaching-related and Student-related.

#### Teaching-related

A number of teaching related issues were reported, including the labour-intensive nature of teaching scalpel skills, the need for teaching consistency, the need for evidence-based competencies and safety (Table 4).

**Table 4:** Frequency of teaching or student related issues regarding teaching manual clinical skills

Issue	Count (*n* = 16)
Staffing/Labour intensive	3
Dexterity	4
Psychological issues	
Confidence	5
Motivation	4
Anxiety	5
Clinical Experience	
Patient Availability	3
Clinical Exposure	6

#### Issues

Teaching podiatric manual clinical skills was identified by many participants as a resource intensive process in comparison to other university programs or subjects. Scalpel use, particularly in early learning or instances of struggling students, was perceived by many interviewees to require high levels of supervision, such as outlined by the following data,

*“…although it is expensive you have to put really low student (to) staff ratios into those preclinical skills to get the students as competent as they can be early on rather than trying to correct things at a later stage”* (Participant 7).

This was also highlighted as an issue directly influenced by the increasing number of students in programs as another participant explained,

*“…there is always going to be issues around that (sic) the class sizes getting bigger…..so monitoring people is always going to be an issue”* (Participant 16).

The podiatry programs in this study reported teacher to student ratios between approximately 1:4 and 1:7 for the relevant clinics.

The perceived need for teaching consistency was also an issue. It’s *“better if one person can teach the skill to the students”* (Participant 14), however if this is not possible then *“consistency is really important…..with an agreed process between all members of staff”* (Participant 4). Yet it was identified that due to the uniqueness or different styles of tutors and educators there were likely to be differences in teaching and that this could cause confusion or difficulties for students, particularly students who were struggling. Differences in teaching were perceived to stem from differences in teaching styles, different emphases, differences in scalpel blade choice, individual differences in the technique of the staff, working in different environments or a difference in expectations (for example between a university-based clinic and external clinic placement). There were also issues around teaching scalpel skills to left-hand dominant students if the staff member was right-handed and presumably vice versa; where possible a like-handed tutor was preferred.

Some participants identified that podiatry lacked suitable evidence-based teaching models, outcome measures and competencies as illustrated by the following statements, there is:

*“…no way of measuring their capabilities, (we) don’t have lots of competencies like nursing, but maybe it’s because we haven’t structured it that way”* (Participant 10)

*“…not a suitable simulation model we can use… wax is hardly sufficient… I mean putting wax on a piece of moleskin is hardly a good model to use….. without them (simulation models) students practically go straight from virtually no experience to practising on live patients”* (Participant 9)

*“…no such thing as an adequate practice dummy…and it doesn’t matter how much effort we put in to allowing them (students) to carve up soap… I think it’s a good introduction but nothing prepares them for a foot that moves…or… that’s got a person attached”* (Participant 16)

Achieving adequate clinical experience for teaching related to patient numbers and exposure was another challenging issue identified by participants,

*“…if we had more patients we’d treat more and get more (experience)… manual skills would obviously improve at a quicker rate”* (Participant 13)

*“we are relying on the space we have, the rotation through of patients and the time we have to allow them to do it”* (Participant 16)

In part, this was suggested to come from the limited time available for students to practice in the clinic or skills laboratories with or without patients, which in turn can be related to staffing issues as mentioned above and the time available to provide supervision. However, participants also related this issue to patient numbers and available infrastructure. This was further compounded by perceived inadequate exposure of students to, or availability of, some clinical conditions as they may only gain clinical experience in what presents *“on the patient, which may be* ad hoc*”* (Participant 12).

As outlined in the reporting for research aim one, many programs used wax, or wax impregnated materials as low fidelity simulation for students to practice. Many participants highlighted that whilst wax models or similar may be useful for initial practice and technique development, it cannot completely replace real skin.

*“practising on real skin works best and I don’t know how you get around that … callus is completely different to fleecy web so try and get them onto real skin as soon as possible but depends if (it is) available on (their) partner”* (Participant 12)

*“one of the students this year said …they would prefer to have actually done (practised) on a patient, because the texture and everything (of the wax) is not the same”* (Participant 5)

However, practising on real skin requires live human participants which may have significant quality of teaching and safety implications. Safety was identified as an issue in the training of scalpel skills by three participants in the context of the need to ensure student safety, guarding against complacency and ensuring students possess the required ability. The following statements relate to this concern:

*“it is dangerous… we did have a big scalpel injury in the past…so guarding against complacency (on behalf of students)”* (Participant 11)

*“we have had it happen twice where we put the wax impregnated fleecy web onto a student and the person doing the debridement has been more vigorous than they should have been”* (Participant 15)

A contrasting opinion regarding the use of models or alternatives to real skin is outlined under successes below.

### Successes

Despite contrasting statements regarding the legitimacy of using inanimate objects as models for practice, many participants reported that the use of models was more time efficient, safer, less intimidating and able to better engage students.

*“rubberised feet are really helping because when they used to sit there and do nothing for half the class they’d get bored, get disruptive but here with this you can really intensify a 3 hour session or how many hours session you have…”* (Participant 1)

*“practising on innate (sic) objects tends to work well to get students competent, a scalpel can be intimidating…”* (Participant 2)

*“the wax on felt works very well… and (we) find that the students are less apprehensive and less anxious if they are taught that way rather than straight onto a foot and the safety issues around that as well’* (Participant 14).

Yet, it was also perceived by some participants that getting students into clinic and a real-life situation early on in the program was beneficial. Therefore, as practice on real patients was inevitable, numerous successful strategies to mitigate risk were identified. This included a targeted selection of low risk patients with less complex lesions for students to practise on early in student learning and using low staff to student ratios.

Other teaching approaches reported as successful reflect some of the current practices outlined previously, including: the use of multimedia, pairing of students for practice or observation, and breaking down the skill into its substituent components for early instruction.

### Student-related

#### Issues

A number of student-related issues which impact on the teaching of scalpel skills were identified. These included psychological factors, general dexterity and issues specific to international students (Table 4).

Scalpels and their potential to inflict harm were considered daunting for students. Psychological factors such as increased student anxiety or lowered confidence, particularly relating to the risk of harm to themselves or the patient were reported as having an impact on the students. For example, participants commented:

*“So when that (treating a patient) actually happens, when they do come across a patient with callus that needs to be debrided, they’ve usually got a peer alongside of them to help them because they’re quite nervous …their anxiety and their fear of haemorrhaging a patient and that (it) seem(s) to be that their general feedback on their reflection is that they are all terrified of actually cutting someone”* (Participant 14).

From a psychological perspective, lowered student motivation may also impact on learning. The lowered motivation may be for any number of reasons: *“…in some cases it’s just that they really don’t want to be there… and that can reflect in poor performance”* (Participant 15).

A lack of dexterity was also a common student-related issue identified, with one participant suggesting they should undertake prerequisite testing for the program prior to admission:

*“It would be lovely if (we) could screen all new people coming in for their ability and if they have (good) manual dexterity skills…. I have been in places in the past where they do a dexterity test”* (Participant 1)

However, one participant commented that they did not think screening was necessary. There was a spectrum of experiences reported relating to the issue of dexterity with some participants describing students presenting with poor dexterity impacting on their long-term prospects, whilst others felt that dexterity is something that could develop in all students over time and that they had never had a student who couldn’t proceed in the program as a direct consequence of poor dexterity. The diversity of opinion on this issue is illustrated by the following contrasting statements:

*“There is occasionally a student (who) has no clue, no idea and no skill so we would encourage that student as much as possible to develop those (skills) but if at the end of the day the student just couldn’t get it, then there has been occasions when we said look, ‘this isn’t really for you’….some people just can’t use a scalpel it doesn’t matter how many times you break it down or change it…”* (Participant 1)

*“The classic is you have students who have done tech(nical) studies, a lot of craftwork or some sort of fairly complex fine motor skills, they tend to be hugely advantaged over students who have had very little or no exposure to those sorts of skills at all..”* (Participant 15)

*“It’s very rarely (sic) that we get people come in to the course that don’t have good hand dexterity, I think they understand pretty quickly that they need to have that..”* (Participant 7)

*“…I think it’s pretty hard to do that at the end of first year, to tell somebody, ‘right, you’re not going to cut it, go and find something else to do’, there are so many other factors… you know it’s our job to make sure that the clinical skills improve over time… when I started out, my clinical skills, my manual skills, callus debridement, you know they just improved and improved every year and I’d say after 10 even; when I’d been at it for 10 years…”* (Participant 13).

International students were highlighted as a group who often experienced difficulty learning manual clinical skills. It was suggested by participants that this could be related to comprehension when English was a second language and not having the confidence to speak up, or that the dual task of talking (to the patient) and undertaking the manual clinical skill was more complex when English was not the student’s first language.

### Managing poor performers

The second category to emerge from the qualitative content analysis relates to how podiatry students who are struggling or performing poorly in the area of manual clinical skills are managed.

#### Strategies

A range of strategies were nominated for managing poor performers, including increased teaching or practical exposure, increased positive feedback (and avoiding negative feedback), and moving students back a step on the teaching continuum to decrease complexity.

*“…or they just might need some I call it a peptalk, but encouragement, to demonstrate that skill without feeling nervous or being worried about harming the patient in that way; and reassurance as well, knowing that it as part of learning process…”* (Participant 14).

*“…if you say anything negative to the student, that’s going to put a block up and they won’t perform well at all, ever”* (Participant 5).

*“…in a lot of cases that may be going back, taking them right back to the simple task of using a piece of soap using a piece of wax impregnated make-believe lesion and then bring them forward again”* (Participant 15)

An increase in teaching or practical exposure to scalpel skills included further demonstration, increased supervision or mentoring, the introduction of further competency checklist requirements, pairing up with stronger students or increasing the time students were exposed to patients.

Each of the university programs reported they employed one, or a combination of the outlined strategies.

## Discussion

This study determined that the approach to teaching manual clinical skills, in particular scalpel skills, appears to be consistent between university programs in Australia and New Zealand, utilising didactic-style content, demonstration, physical practice on inanimate objects and real skin, and often the use of supplementary audio-visual material. There were some differences reported between programs, mainly around the methods and processes of practice. The primary area of controversy regarded the use of inanimate objects versus real skin for practice, which was reflected in comments relating to both issues and successful strategies in teaching.

Most podiatry programs followed a traditional teaching structure when teaching scalpel skills with a didactic-style lecture or talk outlining the theoretical and explicit information relevant to using a scalpel supplemented by skill demonstration, either in real-time, streaming media or via a recorded medium such as DVD or online video. This teaching approach is consistent with evidence that observational learning together with practice is more effective for motor learning than practice alone [12], and is supported by studies which show that similar areas of the brain activate whilst a student is observing a particular skill being performed as when they are undertaking that skill themselves [13–16].

The reported mentoring relationships and strategies of students working in pairs provide an alternative avenue for such observational learning. When working in pairs, students are able to watch someone else undertake a learning process, which is believed to facilitate adaptation when they are required to perform the same action [17–22]. Such observational learning provides the opportunity for students to continue to maximise learning by engaging in alternative forms of processing, whilst having a break from the cognitive demands of the skilled task [20]. Moreover, the non-treating student can take the responsibility of engaging the patient, allowing the practising student to focus their cognitive resources and concentrate on learning and performing the skill. Working in pairs may also have an influence on the motivation and competitiveness students feel for the task, which can have a significant influence on learning outcomes and address psychological issues [18, 20, 22, 23].

From the limited, but growing, body of literature, there is increasing evidence that simulation based practice, combined with deliberate practice is superior to traditional methods [24] and that simulations may not need to be of high fidelity for novices [25–27]. In a systematic review for medical clinical education McGaghie et al. found an effect size of 0.71 (95%CI 0.65–0.76; *P* < 0.001) [24]. However, the individual contribution of simulation versus deliberate practice was not determined. Furthermore, we should be aware of the confounding effects that feedback, motivation and other known factors may have had within the studies comprising the meta-analysis.

The findings of McGaghie et al. were supported by our findings whereby, the role of practice in teaching of manual clinical skills was highlighted by participants from all programs. Practice was often undertaken on inanimate objects and foot models, and perceived as a successful teaching strategy as it reportedly decreased students’ relative anxiety, increased engagement and provided a safer alternative than a real-life patient. Half of the programs reported using specific inanimate objects followed by wax added to some sort of base such as tape or fleecy webbing (wax impregnated materials). All except one program reported using wax on a base, with the remaining program using fleecy web only. In a majority of programs, students were paired-up and the wax was attached to the foot of their partner to enable more context-specific practice. However, there are caveats to this and a contrast of opinions There is an expectation that transfer of learning from laboratory practice to the real setting is automatic [28]. However, the transfer of learning from one skill to another is proportional to the similarity of the skill and may depend on the environmental context [28]. Furthermore, questions remain about how best to structure simulation interventions and the best frequency and timing for effective learning acquisition and skill retention [25].

Conversely, participants reported that it was good to have students put into a real-life situation early as the wax does not sufficiently replicate real skin. It was felt that there is a lack of suitable models. This may be compounded by a lack of sufficient clinical exposure, particularly in the context of growing class sizes and the fact that students and teachers often rely on real patients attending the university podiatry clinic for practice exposure to a variety of conditions. It is for these reasons a combination of practice strategies was often utilised.

The differences in opinion from participants regarding open (real-life) versus closed (simulated) learning is also reflected with much controversy in the literature. This may stem from difficulty interpreting the evidence and difficulty extrapolating findings from skill acquisition research to real life application. Kneebone [29] outlines that this may be a consequence of the limited outcome-based rather than descriptive studies, often with small sample sizes and short intervention periods.

Safety was also a concern that influenced the practice strategy used with students. Practising on real patients creates a paradoxical dilemma where the public are exposed to potential risk associated with receiving treatment from inexperienced students, but with students relying on this exposure in order to gain the required experience. Further, there is controversy in the literature regarding open versus closed skill learning which adds further complexity to choices about student practice in this area, with some authors promoting research which supports open skill learning, such as a real-life clinic with a variable environment [28, 30]. Yet Knight [31], argues that these studies don’t take into consideration the effects of anxiety and arousal which are present particularly in learning many of the health-related clinical skills. Furthermore, Motola et al. outline that learning the whole skill at once rather than being introduced in parts may be detrimental to learning if there is an increased cognitive load [25].

The structure of practice reported by programs did not always reflect the available evidence. Practice of podiatry-related manual clinical skills, particularly scalpel skills occurred in a massed or blocked form in some programs, whilst others used practice intermittently or spaced over an extended time. Whilst there was an early belief that intermittent or spaced practice was the superior approach for learning simple motor tasks, more recent research suggests that outcome effect sizes decrease with increasing task complexity and cognitive demands [32]. The role of feedback, contextual interference and motivation can also have a strong influence. Interpreting studies relating to motor skill acquisition should be undertaken with care, as findings based on simple skills do not take into account the greater cognitive demand and other aspects typical of complex motor skills. Thus, we should recognise that the plethora of research designs, structure and outcomes makes interpretation and synthesis of published data to inform the teaching process quite difficult.

The practice setting and associated safety concerns were important issues recognised by participants in learning manual podiatric skills. A variation in practice environments reported between programs, such as home, supervised clinic and unsupervised clinic has the potential to vary the type and timing of feedback a student receives about their performance of a skill. For instance, the practice environment can influence whether the student receives extrinsic augmented feedback or feedback only via intrinsic mechanisms. Mars et al. [33] found that the part of the brain responsible for error detection and correction was activated by extrinsic feedback earlier in the learning process than intrinsic feedback. Furthermore, it is commonly thought that when augmented feedback is provided too frequently, the learner becomes dependent and no longer able to perform effectively when it is withdrawn [34]. There is debate whether this applies to complex tasks as well as simple tasks [35]. However, it still seems that the exact timing, frequency and type of feedback can have a significant influence on task learning.

Learning may be encouraged through facilitating student autonomy. When students have autonomy over learning, when to practise and when to receive feedback, they display improved motor learning [36–38]. It is believed that this may be related to motivation [22, 39–41], specifically intrinsic motivation. Many of the podiatry programs attempted to do this either intentionally or unintentionally via the use of multimedia or enabling safe ways for students to practise independently.

One of the teaching-related issues in the study findings was the labour-intensive nature of teaching scalpel skills and difficulty fitting this within the University-driven workload model, potentially producing a mismatch between necessary supervision levels and the time availability and priorities of staff. The staff-student ratio specified by the Australian and New Zealand Podiatry Accreditation Council (ANZPAC) is 1:4 to 1:10 depending on associated risk [42]. For example learning scalpel skills in a clinic environment involving novices should have a low ratio, which makes it both labour-intensive and expensive. In addition to staffing ratios, the ANZPAC recommends that 1000 clinical hours need to be achieved by students be eligible for registration. Sixty percent of these hours should be completed within internal university clinical facilities putting further pressures on university staff and workload. However, these time requirements do not specifically take into consideration the patient numbers treated, amount of pathology or variety of pathologies to which the student is exposed, or the competency of a particular student. These factors will also impact on the motor skill capability related to scalpel skills of a particular student.

It is possible that the choice to enforce a clinical time requirement to ensure registration standards are met may stem in-part from a lack of objective outcome indicators and established competencies within the teaching of manual podiatry skills. A point identified by some of the interview participants. Bradshaw [43] reported for the nursing profession that without clearly defined competencies and prescribed assessment standards, graduating students were concerned about their practical skill competence. ANZPAC has developed a collection of competencies for the purpose of evaluating foreign podiatrists seeking registration in Australia, which at least one interviewee reported modifying for use in the undergraduate educational setting. However, it seems that further work in developing relevant podiatric competencies may be beneficial and would help address the issue of teaching consistency also identified.

Despite the identified lack of agreed skill competencies, the most commonly employed assessment method reported was the use of clinical examination in the form of a single examination or as an ongoing requirement for students to be signed-off on particular aspects of competency. Ongoing assessment was more commonly reported to be associated with evaluating a minimum skill threshold rather than grading performance. This potentially has the added benefit of invoking less anxiety in the students, thereby facilitating greater safety for both the students and patients concerned. Also, through the use of an ongoing competency checklist, poor performers can be identified easier and earlier, providing a greater opportunity for remedial strategies to be used.

Motivational states are influential to the learning of complex skills [22] and were identified as a student-related issue in this study. The defining feature of intrinsic motivation is that it needs to be driven by the individual themselves, not just by the educators or other external mechanisms. There are also other student-related psychological aspects which may impact on motor learning, including high levels of anxiety and decreased confidence or self-efficacy. Evidence consistently demonstrates a high correlation between an individual’s self-efficacy, motivation and subsequent performance [44]. Self-efficacy has been linked with motivation [44–46] and anxiety [44–48], itself affecting performance. A number of participants outlined the need to avoid negative comments and instead provide positive comments in an attempt to positively reinforce or increase a student’s self-efficacy. Self-efficacy may mediate anxiety and the effect it has on performance [48, 49]. A number of reported experiments found that self-efficacy was the major predictor of anxiety, even more than the individual’s susceptibility to anxiety [49]. There are consistent findings that managing perceived self-efficacy is of greater benefit than merely managing anticipatory anxiety in avoidance behaviour [49], and therefore may be a more suitable method of managing students learning scalpel use.

A student’s ability to reach a sufficient level of dexterity was identified as an issue in this study with the suggestion that a screening test could be useful, similar to that used by dental programs in North America [50, 51]. However, the importance of screening was not universally agreed by participants. Research undertaken on a number of pre-entry skill aptitude tests for study programs have been shown to have poor correlation or non-significant outcomes between the test and success in the program, associated learning, or subsequent performance [50–56]. This is clearly an area for future research, including consideration of the student perspective.

A number of remedial strategies were discussed to manage poorly performing students, which were targeted at increasing practice and improving dexterity. Participants reported strategies of increasing practice time, increasing exposure to relevant conditions, increasing educator input and feedback, providing reassurance or building the confidence of the student or by pairing struggling students with stronger students to maximise upon the benefits of observational learning discussed earlier. Dexterity is considered a skill which may be learnt over time rather than being entirely innate. There has been little research into the best ways to improve dexterity, particularly in health professionals, and therefore does not address the dilemma where students have a need for increased exposure and practice which may increase the risk to the patients or students.

There are limitations in the use of semi-structured interviews to investigate the teaching approach in podiatry programs. Despite an overall feeling that a particular point is relevant or important, participants may not think of it at the time of the interview or may prioritise other points. Therefore, the findings from this study cannot be considered a ranking of issues nor an exhaustive indication of the breadth of all relevant issues. Furthermore, participants are likely to be influenced by their most recent exposures or experiences and therefore have a specific bias. Furthermore, other intrinsic and extrinsic factors may influence their answers including their years and breadth of experience teaching scalpel skills, or even time pressures they may have faced at the time of the interview. As the interviews were undertaken specifically on podiatry programs within Australia and New Zealand, the findings may not be generalisable beyond this setting.

On occasion, there were small inconsistencies reported between participants from the same university, for example with the motor practice arrangements as shown in Table 2. All views were reported; however, this may reflect a lack of standardised rules regarding scalpel skill teaching within some programs

This study highlights the need for further research into practical skill instruction, in particular scalpel skills in the podiatric student population, the use of simulation and practice arrangements (including temporal aspects) and their translation to real-life practice. To build upon the evidence provided in this article an investigation of the impact on staff or the perception of students regarding issues in scalpel skill learning would be beneficial. Furthermore, a quantitative evaluation of the scalpel skills relating to methods of instruction or those facing the identified issues would be useful for further progress.

## Conclusion

Teaching manual clinical skills, particularly scalpel use, is a complex process that may be affected by a number of influencing factors. Despite a lack of research and literature surrounding this topic, particularly in podiatry, the approach to teaching is relatively consistent between programs in this study, with the greatest disparity being the arrangements around practice structure and duration.

From the perspective of podiatry program teachers, key issues in teaching podiatry-related manual clinical skills were students’ clinical exposure, motivation, levels of anxiety and dexterity. These areas deserve greater attention and research to ensure ongoing improvement in service provision and safety for the public and students.

The findings highlight the particular importance that dexterity and clinical exposure play in the development of scalpel skills, and as such, it is vital for a safer evidence-based approach to be determined, particularly in the instance when struggling students are identified.

## Abbreviations

ANZPAC: Australian and New Zealand Podiatry Accreditation Council
OSCE: Objective Structured Clinical Examination
